# Long noncoding RNA EPB41L4A-AS2 inhibits hepatocellular carcinoma development by sponging miR-301a-5p and targeting FOXL1

**DOI:** 10.1186/s13046-019-1128-9

**Published:** 2019-04-10

**Authors:** Yu-Gang Wang, Tao Wang, Min Shi, Bo Zhai

**Affiliations:** 1 Department of Gastroenterology, Shanghai Tongren Hospital, Shanghai Jiao Tong University School of Medicine, Shanghai, China; 2 Department of Interventional Oncology, Renji Hospital, School of Medicine, Shanghai Jiaotong University, Shanghai, China

**Keywords:** Hepatocellular carcinoma, Noncoding RNA, EPB41L4A-AS2, miR-301a-5p, FOXL1

## Abstract

**Background:**

Hepatocellular carcinoma (HCC) is the major histological type of liver cancer with high morbidity and mortality worldwide. Long noncoding RNAs (lncRNA) has been proved to be associated with various cancer types, while its regulation in HCC is largely unknown.

**Methods:**

To figure out the specific role of lncRNA EPB41L4A-AS2 in HCC. Fluorescence in situ hybridization (FISH) was first used to determine the cellular sublocalization of EPB41L4A-AS2 to determine its primary mode of action. QRT-PCR, Western blot and hematoxylin-eosin staining were then used to measure the expression of genes in cells and tissues. Cell proliferation and invasion assays were performed to determine the effects of EPB41L4A-AS2, miR-301a-5p and FOXL1 on the malignant phenotype of tumor cells. With luciferase reporter assay, the direct interaction between target genes were further confirmed for research on molecular mechanism. Finally, the mice hepatocarcinoma model was also established to disclose the tumor suppressor effects of EPB41L4A-AS2 in vivo.

**Results:**

Here, we have identified a novel lncRNA EPB41L4A-AS2, which is significantly downregulated both in HCC cells and tissues, and plays a negative regulatory role in HCC proliferation and invasion. Mechanistically, cytoplasmic lncRNA EPB41L4A-AS2 functions as an efficient miR-301a-5p sponge, thereby release the expression inhibition of forkhead box L1 (FOXL1). Indeed, lncRNA EPB41L4A-AS2 inhibits proliferation and migration by upregulating FOXL1 expression and FOXL1 was confirmed as a direct target of miR-301a-5p. MiR-301a-5p shows an inverse correlation with EPB41L4A-AS2 expression and was verified as a direct target of EPB41L4A-AS2 as well. Correspondingly, FOXL1 and miR-301a-5p show opposite biological effects in cell proliferation and migration. Moreover, miR-301a-5p overexpression rescued the EPB41L4A-AS2 upregulation induced depression in proliferation, migration and invasion of HCC cells, as well as promotion effect on FOXL1 expression. Also, in vivo experiments proved that EPB41L4A-AS2 suppress tumor growth and extrahepatic metastasis (lung) via the miR-301a-5p-FOXL1 axis.

**Conclusions:**

Taken together, this research revealed a concrete mechanism of lncRNA EPB41L4A-AS2 in HCC, which may serve as a potential biomarkers and novel therapeutic targets for further clinical application.

**Electronic supplementary material:**

The online version of this article (10.1186/s13046-019-1128-9) contains supplementary material, which is available to authorized users.

## Introduction

Liver cancer is a common malignant tumor type with poor prognosis and high mortality [1]. The incidence of primary liver cancer in China is about 466,000 per year, accounting for more than half of the world’s total, and the death toll is about 422,000, second only to gastric cancer and lung cancer [2, 3]. In recent years, progresses have been made in diagnoses and treatments of liver cancer, while the prognosis of it is still poor. The recurrence and metastasis rate of liver cancer is as high as 70% within 5 years after operation [4]. As the main subtype of liver cancer, hepatocellular carcinoma (HCC) is the most frequent type of cancer in men under 60 years of age, with the highest mortality rate in the world [5]. However, the molecular mechanisms of HCC still remain unknown, especially in terms of tumor development. As with various cancers, early detection of HCC has a greatly improved prognosis compared to the advanced stage [6]. Therefore, fields focusing on HCC developments are in great need of identification and profound understanding of related biomarkers and therapeutic targets.

Long noncoding RNAs (lncRNA) are a large component of noncoding RNA, which is longer than 200 nucleotides in length [7]. Their expression shows specificity in various tissue types, and this kind of expression patterns suggests that they may play an indispensable role in concrete pathophysiological processes [7]. In recent years, evidences have demonstrated that lncRNAs regulate in both two sub-regions of cell: chromatin interaction, transcriptional regulation and RNA processing in nucleus; post-transcriptional modification, translation regulation and signaling pathways regulation in cytoplasm [8, 9]. Moreover, emerging studies revealed that lncRNAs participate in almost all processes of HCC development, including tumorigenesis [10], tumor proliferation and metastasis [11, 12], and disease prognosis [13]. Therefore, exploring the cancerigenic or carcinostatic mechanism of lncRNAs in HCC is of great significance for comprehending the etiology and optimizing treatment.

Several classic lncRNAs have been validated as regulatory factors in HCC to date, such as HULC [14], HOTAIR [15] and H19 [16]. Focusing on the role of lncRNAs in cancer development, hypoxia induced downregulated lncRNA-LET induces hypoxia associated cancer cell metastasis through affecting HIF-1α expression and stability by interfering with mRNA [17]. LncRNA ZFAS1 acts as the sponge of miR-150 to upregulate the expression of ZEB1, MMP14 and MMP16, thereby promoting the metastatic behavior of HCC [11]. LncRNA-GIHCG promotes proliferation and metastasis of HCC cells through upregulating trimethylation of histone H3K27 and DNA methylation levels of miR-200b/a/429 promoter by recruiting EZH2 and DNMT1 [18]. However, there are quantities of novel non-classical lncRNAs remaining unexplored, which may show close relationships with development of HCC.

Through quantitative experiments at three levels of cell lines, animal models and pathological tissues, we identified lncRNA EPB41L4A-AS2 which is significantly downregulated in HCC in this study. Previous study has reported that lncRNA EPB41L4A-AS2 shows negative regulatory effect on TGF-β signaling and tumor invasion and metastasis in head and neck squamous cell carcinoma [19]. Therefore, we aimed to explore its underlying molecular mechanism involved in its low-expression in HCC.

## Materials and methods

### Patients and tissue specimens

Nineteen patients were enrolled in this research and all of them were pathologically diagnosed with hepatocellular carcinoma at Shanghai Tongren Hospital from 2010 to 2013. To control the potential confounding factors, patients who received treatments as chemotherapy or radiotherapy were excluded. Tissue samples were immediately frozen in liquid nitrogen at the time of hepatectomy. Study approaches were approved by the Shanghai Tongren Hospital Research Ethics Committee and conformed to the ethical guidelines of the 1975 Declaration of Helsinki (6th revision, 2008), and the informed consents were obtained from all the enrolled patients in accordance with the committee regulations.

### Cell culture

Human hepatocellular carcinoma cell lines SMMC-7721, QGY-7703, the hepatocyte cell line QSG-7701 cells were purchased from the Type Culture Collection of the Chinese Academy of Sciences, Shanghai, China. Cells were cultured at 37 °C with 5% CO_2_ in Dulbecco’s Modified Eagle’s Medium (DMEM, HyClone Laboratories, Logan, UT, USA) supplemented with 0.5% penicillin and streptomycin (Gibco) and 10% fetal bovine serum (FBS, Gibco, USA).

### Construction and transfection of lentiviral vector

In order to overexpress FOXL1, the full-length sequence of FOXL1 was cloned into the pcDNA3.1 vector and then transfected into SMMC-7721 and QGY-7703 cells. The homogenous empty vector was used as negative control simultaneously. FOXL1 3′-UTR reporter plasmid was constructed as follows: the full-length sequence of FOXL1 3′-UTR and the mutated sequences were synthesized and then cloned into NheI and SalI sites downstream of promoter-driven luciferase cassette in the pmirGLO vector (Promega, USA). Similarly, the EPB41L4A-AS2 reporter plasmid was constructed following the above steps.

Lentiviral vector for EPB41L4A-AS2 were constructed by Bio-Link Gene (Shanghai, China). Empty lentiviral vector was also used as negative control. MiR-301a-5p mimic, MiR-301a-5p inhibitor, siRNAs targeting EPB41L4A-AS2 and corresponding controls were synthesized by RiboBio (Guangzhou, China). Lipofectamine 3000 (Invitrogen, USA) was used for the transfection purpose according to the official instructions.

### Cell counting Kit-8 assay

Cell Counting Kit-8 (CCK-8, Dojindo Chemical Laboratory, Kumamoto, Japan) was conducted to measure the cell proliferation. Specifically, a total of 5 × 10^3^ cells were cultured in 96-well plates for 24 h. We then applied the CCK-8 solution reagent to measure the cell viability after transfection treatments. Subsequently, the absorbance was measured at 450 nm after incubating cells with CCK-8 solution (10 μl) at 37 °C for 2 h.

### Transwell migration assay

A total of 2 × 10^4^ SMMC-7721 and QGY-7703 cells with transfection treatments for 48 h were resuspended in DMEM medium (200 μl) and then seeded into the upper chambers of transwell plates (8 μm size, Corning, NY, USA), respectively. After that, 600 μl DMEM medium with 10% FBS was added to the lower chamber. The non-migrated cells were removed after incubation for 24 h at 37 °C. Cells that migrated to the the bottom of the membrane were fixed with paraformaldehyde (4%). After staining with crystal violet (0.1%), cell counting process was carried out with a 200× microscope (Olympus Corporation, Japan).

### Quantitative reverse transcriptionpolymerase chain reaction

Total RNAs were isolated with TRIzol reagent (Life technologies, Carlsbad, CA, USA), and then reversely transcribed with Prime Script RT reagent Kit (Takara Bio, Japan). QRT-PCR analyses were further performed using SYBR® Premix Ex Taq™ (Takara Bio, Japan) in the ABI Prism 7500 Fast Real-Time PCR system (Applied Biosystems, USA). By using 2^-△△Ct^ method, the relative expression levels of the target genes were normalized to that of endogenous control (β-actin). Primers used were listed in Additional file 1: Table S1.

### Western blot

Protein was extracted from cells using RIPA (Radioimmunoprecipitation) Lysis Buffer and then fractionated on 10% sodium dodecyl sulphatepolyacrylamide gels electrophoresis (SDS-PAGE), and transferred to polyvinylidene difluoride (PVDF) membranes (Millipore Corp., USA) with electroblotting. After shaking for blockage with 1 h at room temperature, the membranes were incubated with rabbit anti-FOXL1 polyclonal antibody (1:400) and mouse against β-actin antibody (1:1000) respectively overnight at 4 °C. The membranes were than incubated with horseradish peroxidase (HRP)-conjugated anti-rabbit or anti-mouse IgG secondary antibodies were used at a 1:2000 dilution at room temperature for 1 h. The intensity of visualized signals was accessed with Quantity One 4.6.8 software (Bio Rad, USA).

### Luciferase reporter assay

As Figs. 4f and 6d showed, the mutated reporter was constructed with base deletion of the direct binding region. In the course of luciferase reporter assay, 5 × 10^5^ HEK293T cells were cultured in 24-well plates overnight, and pmirGLO-EPB41L4A-AS2-WT or pmirGLO-FOXL1-WT reporter plasmids (150 ng) and corresponding mutated vectors were cotransfected into cells with miR-301a-5p mimic (50 nM) with Lipofectamine 3000. After cells culture for 36 h, the relative luciferase activities were calculated based on Firefly/Renilla fluorescence with Dual-Luciferase Reporter Assay System according to the instructions.

### Mice hepatocarcinoma model and in vivo study

Mice hepatocarcinoma model was built as reported [20]. Neonatal B6C3F1 mice were injected intraperitoneally with a single dose (6 mg/kg) of AFB1 (Sigma Chemical Co., St. Louis, MO, USA) in 10 μL of DMSO (Sigma) on the 4th day. At the age of 72 weeks, mice were sacrificed and liver tissues were collected.

Same batch of 6-week-old nude mice were randomly divided into two groups. QGY-7703 cells (10^7^) infected with EPB41L4A-AS2 lentivirus and negative control were injected into nude mice, respectively. Tumor volume was calculated every 3 days (tumor volume = 1/2 length×width^2^). After 21 days, the mice were sacrificed and the xenografts were removed. The tumor weight and distant metastasis (lung) were assessed simultaneously. The research programme were approved by the Animal Ethics Committee of Shanghai Tongren Hospital, and the protocol for animal experiments were performed following the National Institute of Health Guide for the Care and Use of Laboratory Animals.

### Bioinformatics analysis

Bioinformatics database (http://www.targetscan.org) was employed for determination of FOXL1 as a potential target for miR-301a-5p.

### Statistical analysis

All the statistical data were expressed as the mean ± standard error from at least three separate repeated experiments and analyzed by using SPSS 23.0 software. Student’s t test or one-way ANOVA was applied for comparisons between experimental groups, and differences with *p* < 0.05 were considered as statistically significant.

## Result

### LncRNA EPB41L4A-AS2 is downregulated in HCC and inhibits proliferation, migration and invasion of HCC cells

**Fig. 1:**
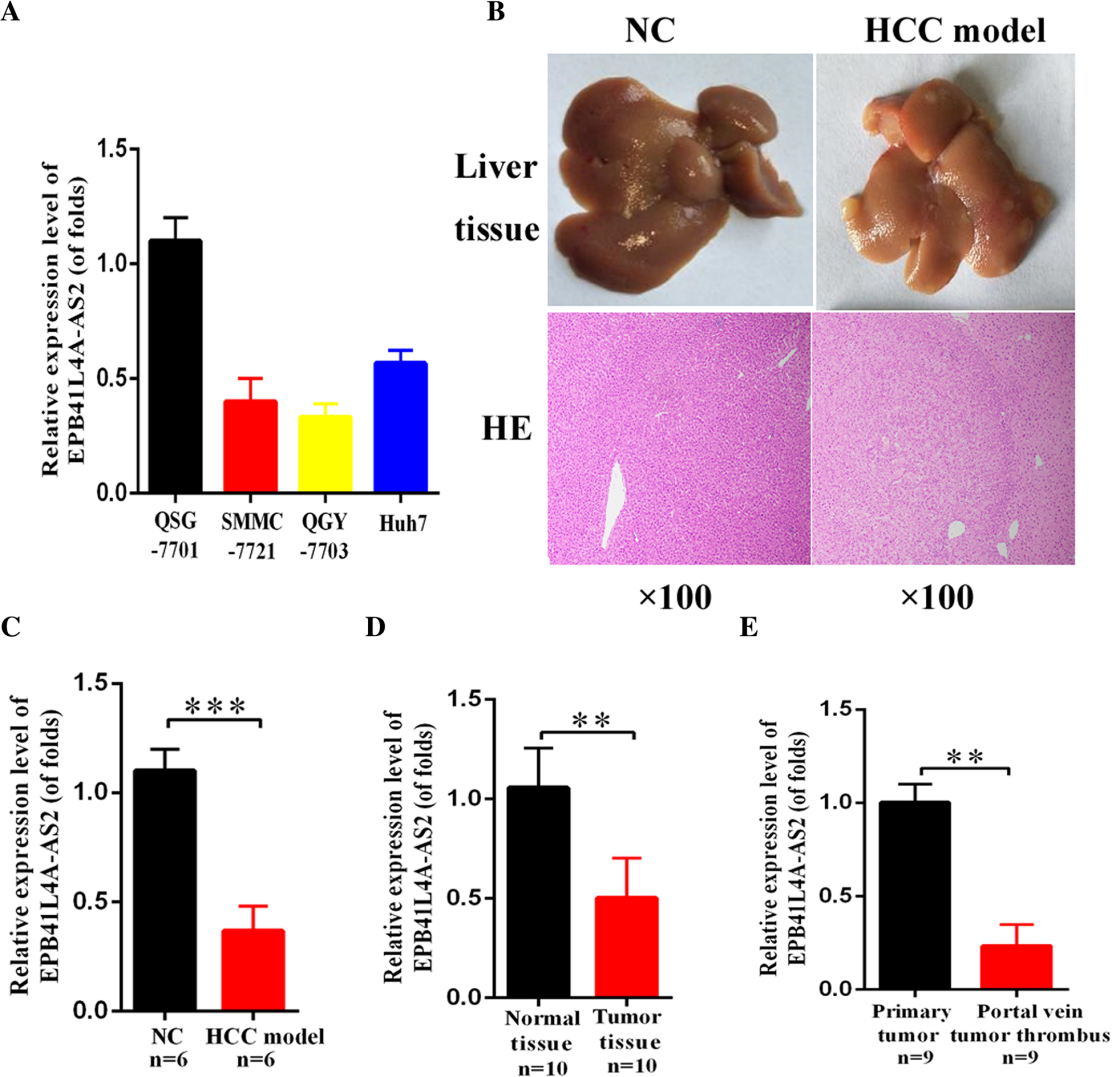
LncRNA EPB41L4A-AS2 is downregulated in hepatocellular carcinoma. **a** EPB41L4A-AS2 is downregulated in hepatocellular carcinoma cell line SMMC-7721, QGY-7703 and Huh7 compared with hepatocyte cell line QSG-7701; **b**-**c** EPB41L4A-AS2 is downregulated in mice hepatocarcinoma model induced by AFB1 (*n* = 6); **d** EPB41L4A-AS2 is downregulated in tissues of hepatocellular carcinoma (*n* = 10); **e** The expression of EPB41L4A-AS2 in portal vein tumor thrombus is lower than that of primary tumor tissues

To comprehensive assess the expression level of EPB41L4A-AS2 in HCC, we analyzed EPB41L4A-AS2 expression by qRT-PCR. Firstly, we explored the EPB41L4A-AS2 expression in HCC cell lines (SMMC-7721, QGY-7703 and Huh7) and the hepatocyte cell line QSG-7701 and found that EPB41L4A-AS2 was downregulated in HCC cells (Fig. 1a). Subsequently, we established a mice hepatocarcinoma model induced by aflatoxin B1 (AFB1) and we found that EPB41L4A-AS2 was downregulated in animal model (*n* = 6) as well (Fig. 1b, c). Moreover, we verified its low expression pattern in pathological tissues of HCC compared with normal tissues (*n* = 10) (Fig. 1d). Furthermore, the expression of EPB41L4A-AS2 in portal vein tumor thrombus (PVTT) was significantly decreased compared with primary tumor tissue (*n* = 9) (Fig. 1e).

**Fig. 2:**
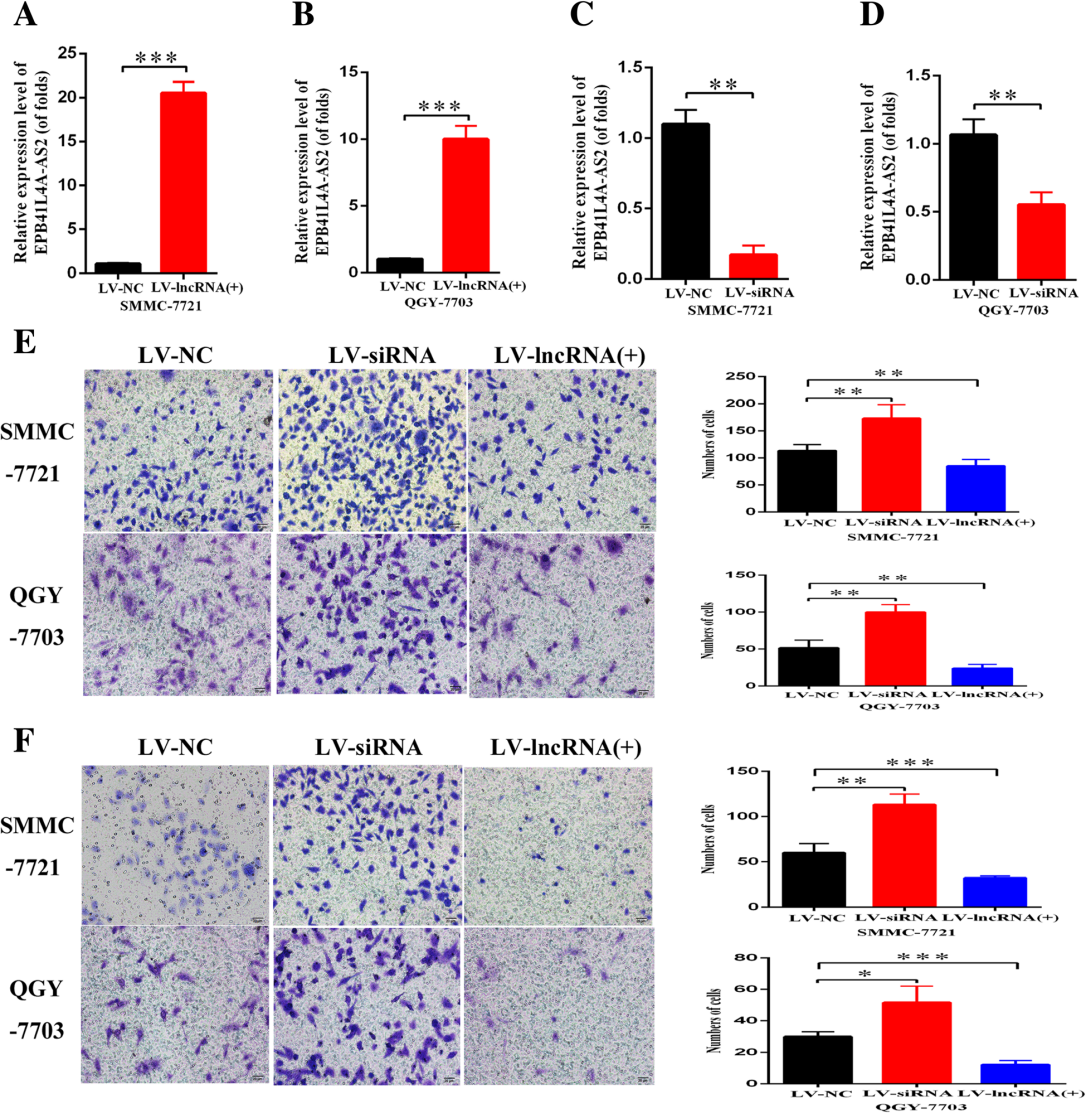
EPB41L4A-AS2 inhibited invasion and migration of hepatocellular carcinoma cells in vitro. **a**-**d** The efficiencies of overexpression and interference for EPB41L4A-AS2 were determined by qRT-PCR; **e**-**f** Transwell assay with or without matrigel showed that EPB41L4A-AS2 decreased cell invasion and migration in SMMC-7721 and QGY-7703 cells

**Fig. 3:**
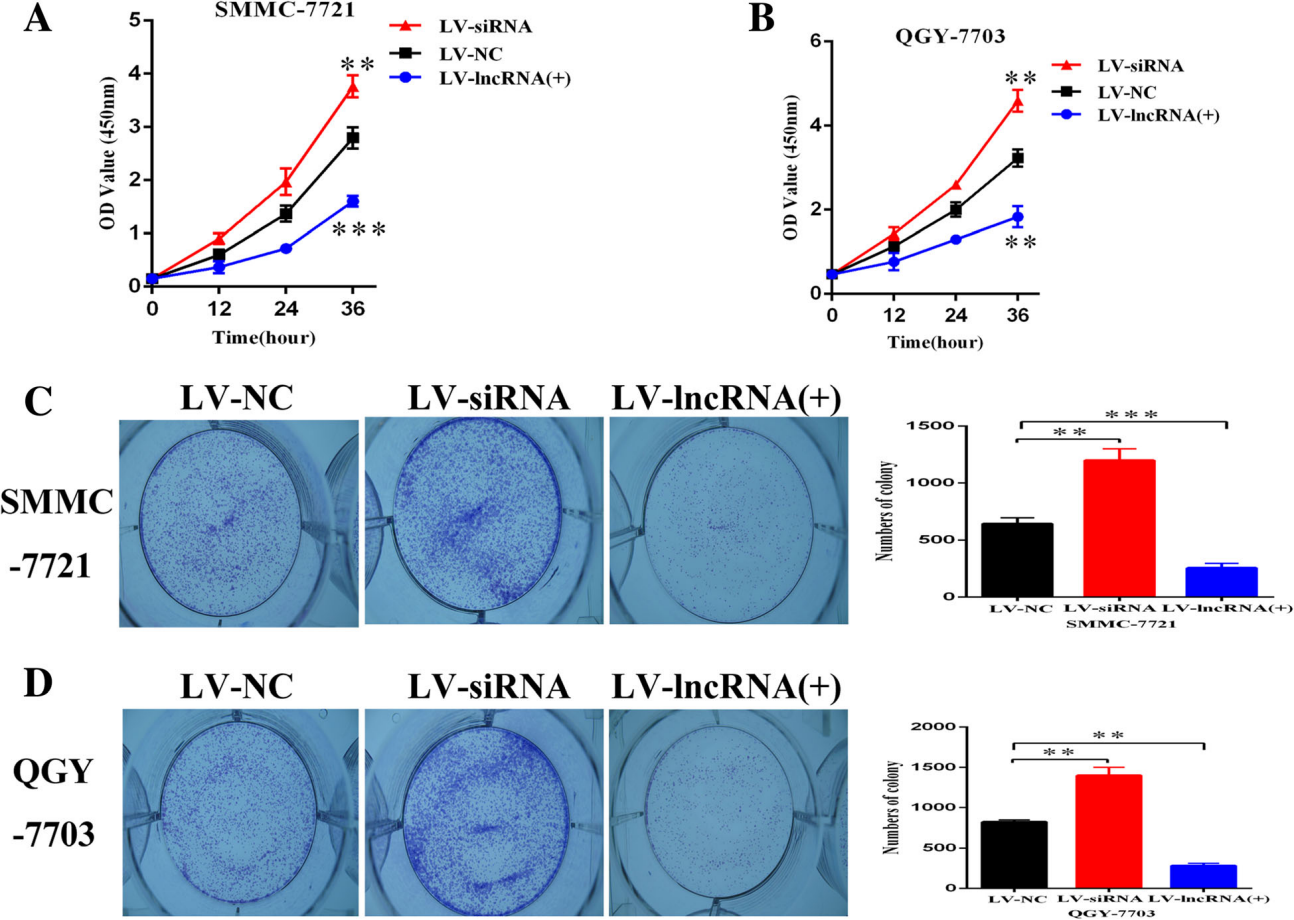
EPB41L4A-AS2 inhibited proliferation of HCC cells in vitro. **a**-**b** CCK8 assays indicated that upregulated EPB41L4A-AS2 inhibited proliferation of SMMC-7721 and QGY-7703 cells, while downregulated EPB41L4A-AS2 played an opposite role; **c**-**d** Colony formation was applied to measure cell viability of SMMC-7721 and QGY-7703 cells

In order to further clarify the biological functions of EPB41L4A-AS2 in HCC cells, we conducted knockdown and overexpression assays in SMMC-7721 and QGY-7703 cells and validated the corresponding efficiency in the both cell lines by qRT-PCR (Fig. 2a-d). Transwell and invasion assays have demonstrated that the downregulated EPB41L4A-AS2 significantly promoted HCC cell migration and invasion. However, the upregulation of EPB41L4A-AS2 conducted by lentivirus transfection dramatically suppressed migration and invasion of HCC cells, respectively (Fig. 2e-f). Meanwhile, CCK8 and colony formation assays revealed that EPB41L4A-AS2 had significant negative regulation effects on cell proliferation abilities in SMMC-7721 and QGY-7703 cells (Fig. 3a-d).

### LncRNA EPB41L4A-AS2 mainly locates in the cell cytoplasm

**Fig. 4:**
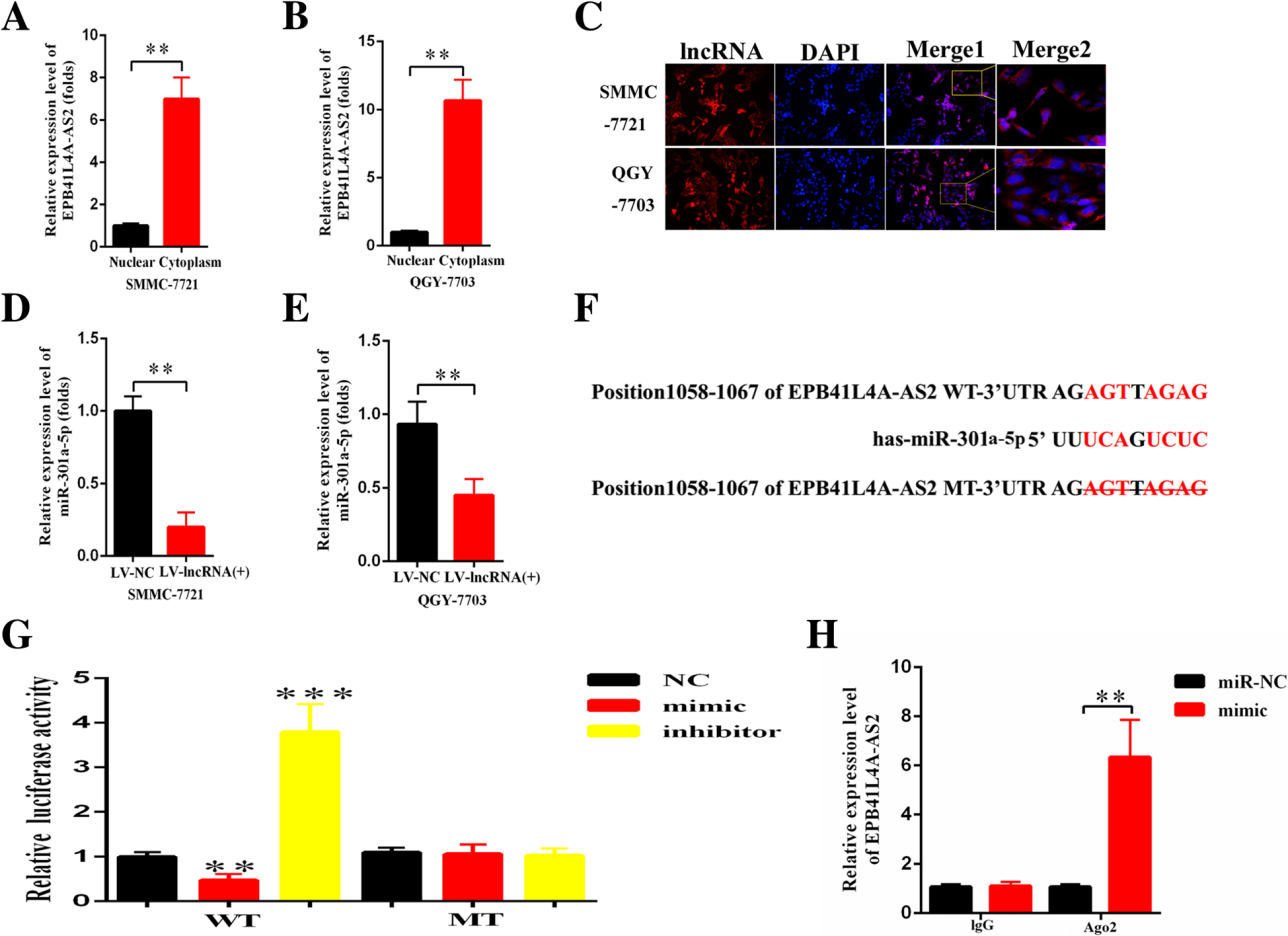
MiR-301a-5p was determined as a direct target of EPB41L4A-AS2. **a**-**c** FISH and qRT-PCR results demonstrated that EPB41L4A-AS2 was mainly expressed in the cytoplasm of SMMC-7721 and QGY-7703 cells; **d**-**e**. The expression of EPB41L4A-AS2 and miR-301a-5p is negatively correlated in HCC cells; **f** The direct binding sites between miR-301a-5p and EPB41L4A-AS2; **g** Luciferase reporter assay for confirmation of direct binding relationship between miR-301a-5p and EPB41L4A-AS2 was performed with luciferase reporter plasmids of wild type EPB41L4A-AS2 and mutant EPB41L4A-AS2. **h** The association between EPB41L4A-AS2, miR-301a-5p and Ago2 was ascertained by analyzing cell lysates using RNA immunoprecipitation with Ago2 antibody. Real-time PCR was applied to detect the change in EPB41L4A-AS2 expression level in the substrate of RIP assay in miR-301a-5p-overexpressed HCC cells

In general, subcellular localization of lncRNA determines the main mode of action, which provides clues for potential functions of EPB41L4A-AS2. Therefore, RNA FISH and subcellular fractionation assays were performed and displayed that EPB41L4A-AS2 mainly located in the cell cytoplasm rather than nucleus. To be more specific, EPB41L4A-AS2 labeled by red fluorescence could mainly be seen in cell cytoplasm (Fig. 4c). Quantificationally, the content of EPB41L4A-AS2 in the cytoplasm is around 7 times that in the nucleus of SMMC-7721 cells, and approximately 11 times in QGY-7703 cells (Fig. 4a, b).

### MiR-301a-5p was a direct target of EPB41L4A-AS2

Considering the distribution characteristics of EPB41L4A-AS2 and its significant downregulated in HCC, we hypothesized that EPB41L4A-AS2 act as a tumor suppressor partly by regulating miRNA action through sponge-like binding. We identified that EPB41L4A-AS2 might sponge with miR-301a-5p via bioinformatics analysis (Fig. 4f). We therefore quantified the expression of miR-301a-5p in SMMC-7721 and QGY-7703 cells with EPB41L4A-AS2 over-expression treatment. As Fig. 4d and e showed, the expression of miR-301a-5p was negatively correlated with that of EPB41L4A-AS2. Furthermore, luciferase reporter assay was conducted to validate the correlation between EPB41L4A-AS2 and miR-301a-5p. Compared with the cells cotransfected with pmirGLO-EPB41L4A-AS2-WT and miR-301a-5p inhibitor, there is a significant reduction in luciferase activity of HEK293T cells with cotransfection of pmirGLO-EPB41L4A-AS2-WT and miR-301a-5p mimic. Meanwhile, there is no marked change of luciferase activity in HEK293T cells cotransfected with pmirGLO-EPB41L4A-AS2-MT and miR-301a-5p mimic or inhibitor when compared with negative control (Fig. 4g). Moreover, RIP experiments showed that EPB41L4A-AS2 and miR-301a-5p were obviously enriched in Ago2-containing microribonucleoproteins (miRNPs) relative to control IgG (Fig. 4h).

### Inhibition of miR-301a-5p restrained the malignant phenotypes of HCC cells

**Fig. 5:**
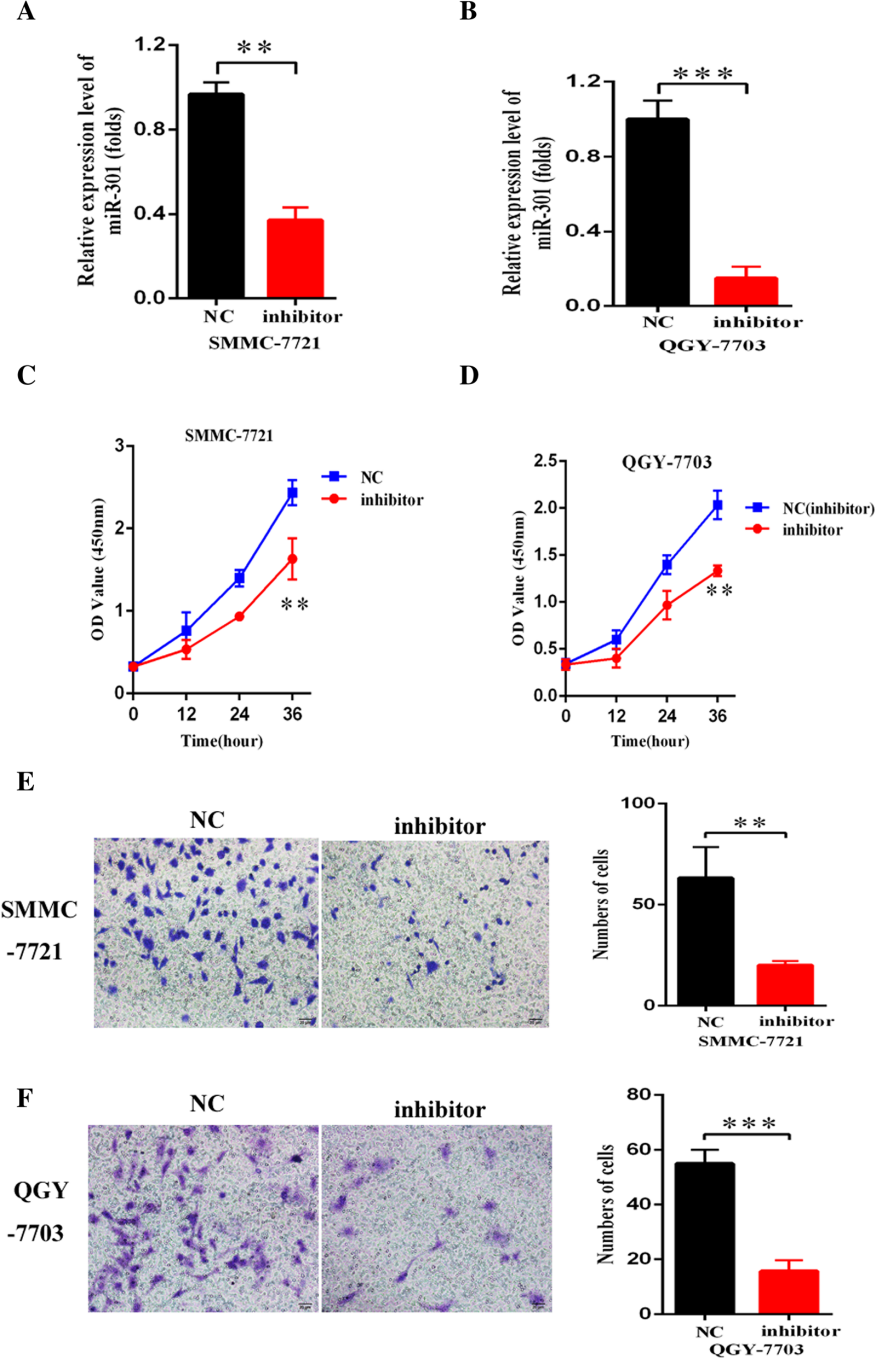
Inhibition of miR-301a-5p restrained the growth and invasion of HCC cells. **a**-**b** The inhibition efficiency of miR-301a-5p expression determined by qRT-PCR; **c**-**d** CCK8 assays indicated that miR-301a-5p suppressed proliferation of SMMC-7721 and QGY-7703 cells; **e**-**f** Transwell assay showed that downregulated miR-301a-5p inhibited cell invasion and migration of HCC cells

In order to further prove the interaction of miR-301a-5p with EPB41L4A-AS2, we explored its influence on the malignant phenotype. Specific inhibitor were synthesize and validated to significantly downregulate the expression of miR-301a-5p in the both cell lines by qRT-PCR (Fig. 5a, b). Cell proliferation assays suggested that downregulated miR-301a-5p had promotive effects on cell proliferation capabilities in SMMC-7721 and QGY-7703 cells (Fig. 5c, d). In addition, transwell and invasion assays demonstrated that inhibition of miR-301a-5p significantly suppress HCC cell migration and invasion respectively (Fig. 5e, f). Taken together, the above data suggested that EPB41L4A-AS2 can inhibit the proliferation, migration and invasion of HCC cells by targeting miR-301a-5p.

### FOXL1 was confirmed as a direct target gene of miR-301a-5p

**Fig. 6:**
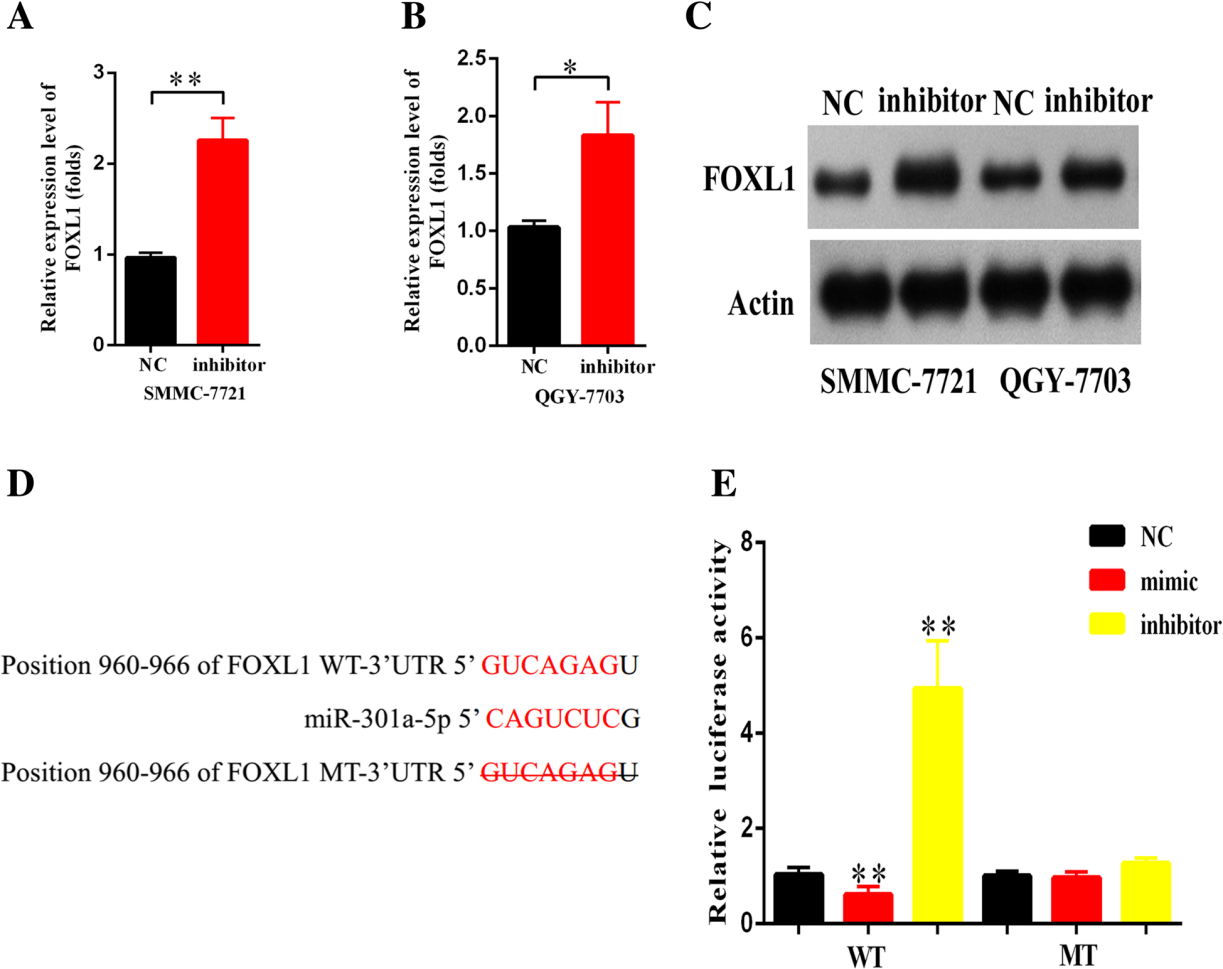
FOXL1 was a direct target gene of miR-301a-5p. **a**-**c**. The expression of FOXL1 and miR-301a-5p is negatively correlated in HCC cells with western blot and qRT-PCR. **d** The binding region between miR-301a-5p and FOXL1; **e** Luciferase reporter assay for confirmation of direct binding relationship between FOXL1 and miR-301a-5p was performed with luciferase reporter plasmids of wild type FOXL1 and mutant FOXL1

To investigate the role of miR-301a-5p in HCC, qRT-PCR and western blot were conducted to confirm the upregulation of FOXL1 in both cells with miR-301a-5p inhibit treatment (Fig. 6a-c). Moreover, we predicted miR-301a-5p targets basing on the miRanda database. As shown in Fig. 6d, the 3′-UTR of FOXL1 contains a putative binding site of miR-301a-5p. Luciferase reporter assay was also carried out to validate the direct interaction between FOXL1 and miR-301a-5p. As shown in the Fig. 6e, HEK293T cells cotransfected with pmirGLO-FOXL1-WT and miR-301a-5p mimic displayed much weaker luciferase activity than cotransfected with pmirGLO-FOXL1-WT and miR-301a-5p inhibitor. While transfection with miR-301a-5p mimic or inhibitor showed no obvious impact on luciferase activity of HEK293T cells transfected with pmirGLO-FOXL1-MT. The above data confirmed that FOXL1 is a direct target for miR-301a-5p.

### EPB41L4A-AS2 inhibited the malignant phenotypes of HCC cells via the miR-301a-5p-FOXL1 axis

**Fig. 7:**
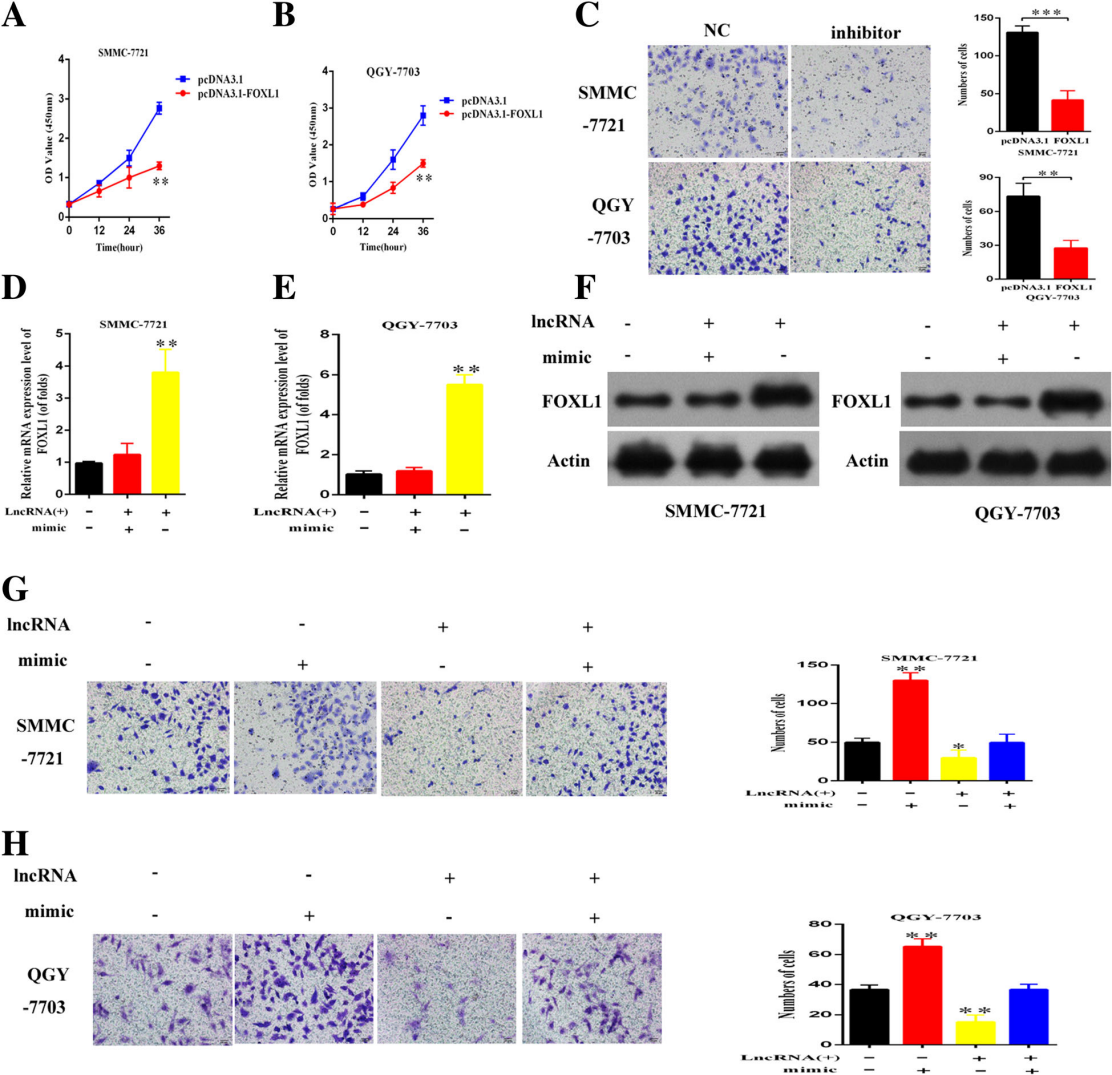
EPB41L4A-AS2 increased FOXL1 expression and inhibited invasion by decreasing miR-301a-5p in vitro. **a**-**c** Upregulated FOXL1 inhibits proliferation, migration and invasion of SMMC-7721 and QGY-7703 cells; **d**-**e** Overexpression efficiency of miR-301a-5p in both HCC cells using miRNA mimics; **f**-**h** Expression of FOXL1 in HCC cells transfected with LV-EPB41L4A-AS2 or miR-301a-5p mimic at mRNA and protein levels; **i**-**l** Rescue experiments by assessing changes in invasiveness of HCC cells in cotransfection with different combinations

After clarifying the sponge-like interaction between EPB41L4A-AS2 and miR-301a-5p, we further investigate whether EPB41L4A-AS2 regulated proliferation and migration of SMMC-7721 and QGY-7703 cells by targeting FOXL1 via sponging with miR-301a-5p. Firstly, cell function experiments indicated that upregulated FOXL1 can dramatically inhibit the proliferation, migration and invasion of HCC cells (Fig. 7a-c). We then overexpressed EPB41L4A-AS2 and found that both mRNA and protein levels of FOXL1 were enhanced. To further uncover intermediate role of miR-301a-5p in EPB41L4A-AS2-FOXL1 axis, we subsequently performed rescue experiments. The ideal transfection efficiency of miR-301a-5p in both HCC cells using miRNA mimics was shown in Fig. 7d,e. The up-regulation of FOXL1 by EPB41L4A-AS2 could be rescued with miR-301a-5p mimic both in mRNA and protein level (Fig. 7f-h). Furthermore, EPB41L4A-AS2 overexpression induced decline in invasion and migration of SMMC-7721 and QGY-7703 cells could also be rescued by miR-301a-5p mimic (Fig. 7i-l). The above data confirmed that EPB41L4A-AS2 regulates the malignant phenotypes of HCC cells via the miR-301a-5p-FOXL1 axis.

### EPB41L4A-AS2 suppressed HCC proliferation and metastasis in vivo

**Fig. 8:**
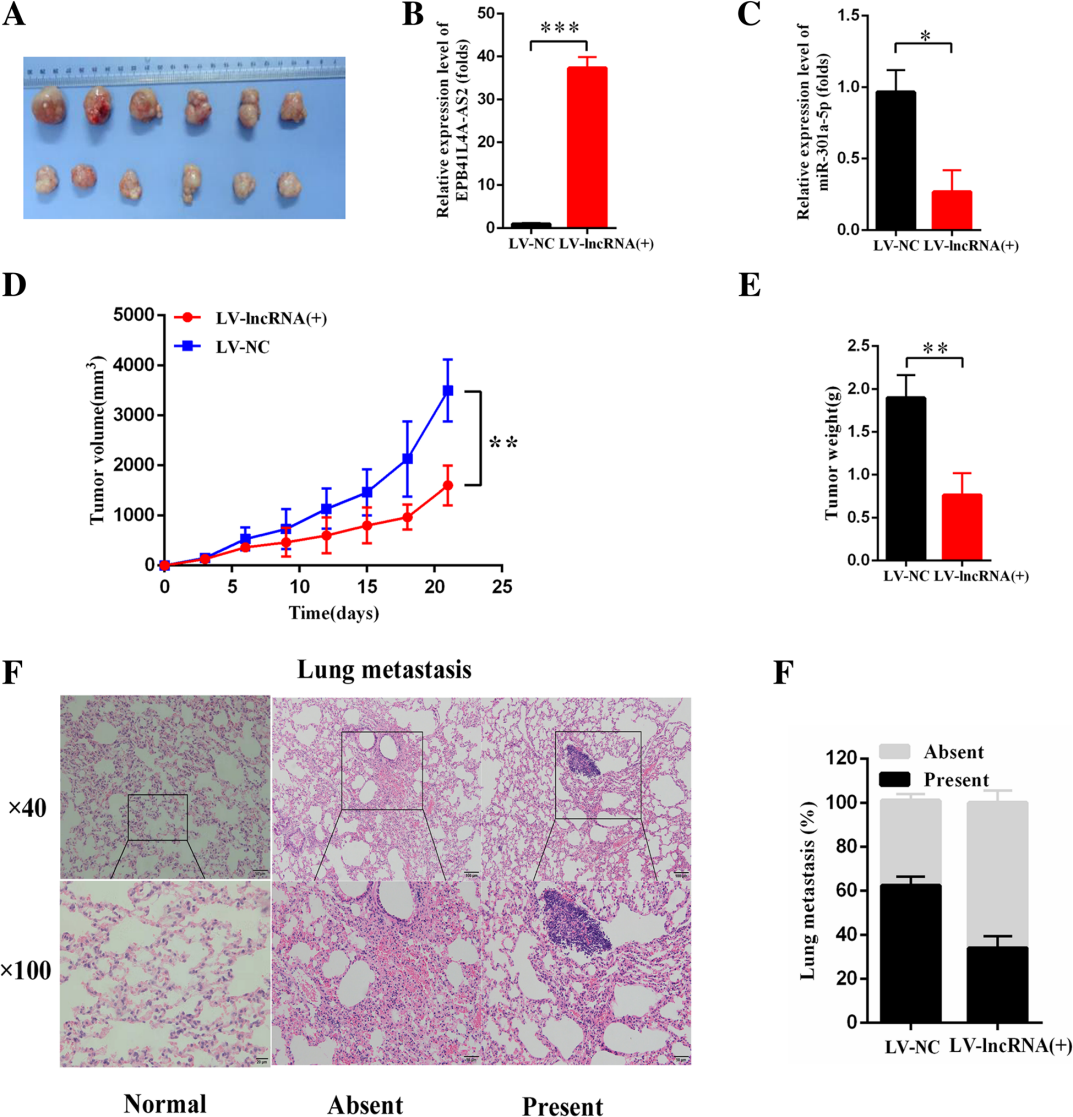
Effects of EPB41L4A-AS2 on HCC cells proliferation and metastasis in vivo. **a** The mice hepatocarcinoma model construction with QGY-7703 cells infected with LV-EPB41L4A-AS2 (*n* = 6). **b**-**c** Expression detection of EPB41L4A-AS2 and miR-301a-5p in xenografts; **d** Time-related volume measurement on xenografts; **e** Final weight evaluation of xenografts; **f**-**g** Calculation on the percentage of mice with or without lung metastasis

In vitro study has shown that the upregulated EPB41L4A-AS2 suppressed the proliferation, invasion and migration of SMMC-7721 and QGY-7703 cells. We therefore speculated that EPB41L4A-AS2 could play the similar role in vivo. Specifically, QGY-7703 cells with stable overexpressed EPB41L4A-AS2 (LV-EPB41L4A-AS2) and that infected with control vector (LV-NC) were used for construction of mice hepatocarcinoma model. The tumor volume was then measured every 3 days for 7 times, and the xenografts were then removed for further analyses (Fig. 8a). We first detected the expression of EPB41L4A-AS2 and miR-301a-5p in the tumor tissue of mice model. As a result, EPB41L4A-AS2 was significantly upregulated and negatively correlated with the expression of miR-301a-5p (Fig. 8b, c). Based on the measurements of tumor volume and weight, EPB41L4A-AS2 amplification restrained the QGY-7703 cells xenograft growth in vivo, which is in accordance with in vitro experimental results. In addition, we indirectly evaluate the effect of EPB41L4A-AS2 on the invasion and migration abilities of QGY-7703 cell by counting the incidence of lung metastasis in mice hepatocarcinoma model. As shown in Fig. 8f, g, lung metastasis decreased in mice hepatocarcinoma model constructed using QGY-7703 cells with high expression of EPB41L4A-AS2. In summary, the biological effects of EPB41L4A-AS2 in vivo and in vitro are relatively consistent.

## Discussion

Hepatocellular carcinoma (HCC) is the main histological subtype of primary liver cancer, which is the third leading cause of cancer death and the sixth most common cancer in the world [21, 22]. Only about 20 to 30% of HCC patients are diagnosed at an early stage, whereas more than 70% of HCC patients have progressed to unresectable disease at the time of diagnosis and the overall prognosis is extremely poor [23]. Therefore, the molecular mechanisms among development of HCC are urgent to be elucidated.

The function of a large number of non-coding sequences in the genome has been receiving much attention [24]. Current researches have found that long non-coding RNA plays an important role in physiological metabolism and regulation [25–27], and its abnormal expression profile has close relationships with HCC developments [28].

EPB41L4A-AS2, a TGF-β-associated lncRNA, has been reported to be associated with the malignant phenotype of tumors and has a potential “sponge” relationship with miR-301a-5p [19, 29]. Simultaneously, we found that miR-301a-5p is not only related to the survival of hepatocellular carcinoma, but also may participate in the development of hepatocellular carcinoma [30, 31]. Currently, we have identified that lncRNA EPB41L4A-AS2 was downregulated in both HCC cells and tissues, and it suppressed the proliferation, migration and invasion of HCC cells. Simultaneously, the low expression of EPB41L4A-AS2 in portal vein tumor thrombus further confirmed its association with poor prognosis of HCC on the other hand. It is actually consistent with previous reports that EPB41L4A-AS2 plays a role as a tumor suppressor in various solid tumors and is associated with prognosis [32]. However, the specific molecular mechanism of EPB41L4A-AS2 in HCC has not been elucidated.

Generally, cytoplasmic lncRNAs can function as sponges of miRNAs, resulting in further arrest of mRNA expression. HOXA11-AS functioned as a sponge for miR-1297 and then antagonized its inhibitory effect on EZH2 protein translation [33]. The competitive binding of lncRNA-PAGBC and tumour suppressive miR-133b and miR-511 are required for its ability on promoting tumour growth and metastasis and activating the AKT/mTOR pathway [34]. In line with this, we validated a novel EPB41L4A-AS2-miR-301a-5p-FOXL1 axis in HCC with application of bioinformatics and molecular biotechnology. As a downstream target of the axis, FOXL1 is reported as an inhibitory role in pancreatic tumor progression through regulation of TNF-related apoptosis-inducing ligand (TRAIL) and zinc finger E-box-binding homeobox 1 (ZEB1, [35]). Moreover, FOXL1 inhibited the proliferation, invasion, and migration of breast cancer both in vitro and in vivo through blocking the Wnt/β-catenin signaling pathway [36].

Here, we demonstrated the negative regulatory effects of FOXL1 in mediating malignant phenotypes as proliferation and invasion in HCC cells as well. Conversely, miR-301a-5p was negatively correlated with FOXL1 no matter in terms of expression level or effects on cell phenotype. Besides, in vitro experiments have also shown that EPB41L4A-AS2 overexpression promotes FOXL1 expression at mRNA level, while the cotransfection of miR-301a-5p lower the FOXL1 mRNA expression. Using bioinformatics and luciferase reporter assay, we have further confirmed the direct binding sites and point-to-point regulatory relationships among the three of them. Specifically, EPB41L4A-AS2 reduces the expression of miR-301a-5p by sponge effect, through the classical ceRNA regulatory mechanism, and then blocks the translation inhibition of FOXL1 by miR-301a-5p. And the development of HCC was suppressed through elevated FOXL1. Additionally, mice hepatocarcinoma model was constructed to further determine the expression pattern and tumor suppressor effect of EPB41L4A-AS2 in vivo.

## Conclusion

In this study, we confirmed our conjecture that EPB41L4A-AS2 regulates downstream FOXL1 through sponge-like binding with miR-301a-5p, thus affecting the developments of HCC. Taken together, this research revealed a concrete mechanism of lncRNA EPB41L4A-AS2 in HCC, which may serve as a potential biomarkers and novel therapeutic targets for further clinical application.

## Additional file

Additional file 1: **Table S1.** Primers designed for qRT-PCR validation of the target genes. (DOCX 15 kb)

## Abbreviations

CCK-8: Cell Counting Kit-8
FISH: Fluorescence in situ hybridization
FOXL1: Forkhead box L1
HCC: Hepatocellular carcinoma
LncRNA: Long noncoding RNA
PVTT: Portal vein tumor thrombus
QRT-PCR: Quantitative reverse transcriptionpolymerase chain reaction
TRAIL: TNF-related apoptosis-inducing ligand
