# Supplementary material for: Long noncoding RNA EPB41L4A-AS2 inhibits hepatocellular carcinoma development by sponging miR-301a-5p and targeting FOXL1
Source: J Exp Clin Cancer Res. 2019 Apr 10;38:153. doi: 10.1186/s13046-019-1128-9 (PMC6458726; doi:10.1186/s13046-019-1128-9)
Supplement: Supplementary file 1 — Table S1. Primers designed for qRT-PCR validation of the target genes. (DOCX 15 kb) [file 13046_2019_1128_MOESM1_ESM.docx]

| Gene ID | Forward primer | Reverse primer | Tm(℃) |
| --- | --- | --- | --- |
| EPB41L4A-AS2 | 5′-GTCGCAGTTAGGGGAGAC  AC-3′ | 5′-TGGCTACCCAGCTAACAAGC-3′ | 60 |
| miR-301a-5p | 5'-ACACTCCAGCTGGGCAGTGCAATAGTATTGTC-3' | 5'-CTCAACTGGTGTCGTGGA-3' | 60 |
| FOXL1 | 5'-GCCTCGCCCATGCTGTATC-3' | 5'-CGTTGAGCGTGACCCTCTG-3' | 60 |
| U6 | 5'-GCTTCGGCAGCACATATACTAAAAT-3' | 5'-CGCTTCACGAATTTGCGTGTCAT‑3' | 60 |

**Supplementary Table 1:** Primers designed for qRT-PCR validation of the target genes
